# Seven novel glucose-6-phosphate dehydrogenase (G6PD) deficiency variants identified in the Qatari population

**DOI:** 10.1186/s40246-021-00358-9

**Published:** 2021-10-07

**Authors:** Shaza Malik, Roan Zaied, Najeeb Syed, Puthen Jithesh, Mashael Al-Shafai

**Affiliations:** 1 Department of Biomedical Sciences, College of Health Sciences, QU Health, Qatar University, Doha, Qatar; 2 Liggins Institute, The University of Auckland, Auckland, New Zealand; 3 Applied Bioinformatics Core, Sidra Medicine, Doha, Qatar; 4 Hamad Bin Khalifa University, Doha, Qatar

**Keywords:** *G6PD* deficiency, Whole-genome sequencing (WGS), Novel variants, Qatar Biobank (QBB), Qatar Genome Programme (QGP)

## Abstract

**Background:**

Glucose-6-phosphate dehydrogenase deficiency (G6PDD) is the most common red cell enzymopathy in the world. In Qatar, the incidence of G6PDD is estimated at around 5%; however, no study has investigated the genetic basis of G6PDD in the Qatari population yet.

**Methods:**

In this study, we analyzed whole-genome sequencing data generated by the Qatar Genome Programme for 6045 Qatar Biobank participants, to identify G6PDD variants in the Qatari population. In addition, we assessed the impact of the novel variants identified on protein function both in silico and by measuring G6PD enzymatic activity in the subjects carrying them.

**Results:**

We identified 375 variants in/near *G6PD* gene, of which 20 were high-impact and 16 were moderate-impact variants. Of these, 14 were known G6PDD-causing variants. The most frequent G6PD-causing variants found in the Qatari population were p.Ser188Phe (*G6PD* Mediterranean), p.Asn126Asp (G6PD A +), p.Val68Met (G6PD Asahi), p.Ala335Thr (G6PD Chatham), and p.Ile48Thr (G6PD Aures) with allele frequencies of 0.0563, 0.0194, 0.00785, 0.0050, and 0.00380, respectively. Furthermore, we have identified seven novel *G6PD* variants, all of which were confirmed as G6PD-causing variants and classified as class III variants based on the World Health Organization’s classification scheme.

**Conclusions:**

This is the first study investigating the molecular basis of G6PDD in Qatar, and it provides novel insights about G6PDD pathogenesis and highlights the importance of studying such understudied population.

**Supplementary Information:**

The online version contains supplementary material available at 10.1186/s40246-021-00358-9.

## Background

Glucose-6-phosphate dehydrogenase (G6PD) is an omnipresent cytosolic enzyme that has an important housekeeping role in all cells. In red blood cells (RBCs), nicotinamide adenine dinucleotide phosphate (NADPH) is produced mainly by the action of G6PD in the first step of the pentose phosphate pathway [1]. NADPH, among other cellular functions, is particularly important in preventing the buildup of reactive oxygen species [2]. Normal activity of G6PD thus helps protect RBCs from oxygen-derived oxidative stress [3]. Gluscose-6-phosphate dehydrogenase deficiency (G6PDD) patients might develop symptoms after exposure to compounds that trigger oxidative stress in RBCs (favism and drug-induced hemolytic anemia), and it is inherited as X-linked recessive phenotype [4]. The WHO classifies G6PDD-causing variants into five classes: class I is the most severe causing chronic non-spherocytic hemolytic anemia (G6PD activity < 1%), class II is also considered severe and associates with acute hemolytic anemia (G6PD activity < 10%), class III is considered as moderate deficiency and it is associated with occasional acute hemolytic anemia (G6PD activity of 10–60%), while classes IV and V are asymptomatic (G6PD activity of 60–150% and > 150%, respectively) [5].

G6PDD affects around 400 million people globally making it the most common human enzymopathy [6]; it is particularly common in the Middle East with prevalence rates reaching up to 39.8% and 30% in Saudi Arabia and Syria, respectively [7, 8]. Over 217 variants of *G6PD* have been reported worldwide, the vast majority of which are point mutations [9]. The most common variants among Arabs are p.Ser188Phe (*G6PD* Mediterranean), p.Ile48Thr (*G6PD* Aures), p.Asn126Asp (rs1050829), and p.Val68Met (*G6PD* Asahi) [10]. However, no studies addressed the molecular basis of G6PDD in Qatar. In this study, we investigated G6PDD-causing variants in the Qatari population using whole-genome sequencing (WGS) data from Qatar Genome Programme (QGP) for 6,045 Qatar Biobank (QBB) participants [11].

## Materials and methods

### Study participants

The study subjects consisted of 6045 QBB participants, and these subjects are Qatari nationals or long-term residents of Qatar (at least 15 years). Participants are aged 18 years or above, and they appeared phenotypically healthy. This includes 3403 females and 2642 males. Detailed phenotypic and lifestyle data were available for the study participants including lifestyle and food intake questionnaires as well as biochemical tests. However, no data were available on the G6PD activity levels. All participants provided informed consent.

### Genetic data

WGS was conducted as part of the Qatar Genome Programme using the Illumina HiSeq TenX platform to an average coverage of 30X. Raw sequencing reads were converted to paired FASTQ format using the bcl2fastq software from Illumina [12], and fastq files were aligned against the reference genome sequence (GRCh37) using bwakit (v. 0.7.11). Variant calling was performed using GATK 3.4, and then, individual vcf’s were undergone for joint calling step to transform into multi-sample VCF file for all participants. After performing the VQSR step, only PASS variants were further used for downstream analysis. Variant calling and filtering steps were performed following GATK best practices [13]. All variants were described in relation to coding DNA reference sequence NM_001042351.3, NM_000402.4, or NM_001042351.1 (specified where relevant). Principal component analysis plot for the genomes used in this study is given in supplementary material, Additional file 1: Figure S1.

### Variant annotation

The variants identified were annotated using SnpEff (SnpEff/SnpSift (v4.3t), which classified variants as high-, moderate-, low-, or modifier-impact variants based on their potential impact at the protein level [14]. High-impact variants include structural, nonsense, frameshift, loss of start codon variants as well as splice site donors/acceptors variants, while moderate-impact variants include 3’ and 5’ UTR variants, exon loss variants, missense variants, conservative in-frame deletions, and conservative in-frame insertions. Low-impact variants include synonymous variants, stop-retain variants, 5’-UTR-premature-start codon gain variants, and splice region variants, while the modifier-impact variants include variants in the flanking regions of the genes, intronic regions, and non-coding regions [14]. Here, we focused on studying the high- and moderate-impact variants, since they are likely to affect the protein function.

### Assessment of the high- and moderate-impact variants

High- and moderate-impact variants were annotated using different databases such as Human Gene Mutation Database (HGMD) and ClinVar. They were also assessed through pathogenicity prediction tools including sorting intolerant from tolerant (SIFT), Polyphen, combined annotation-dependent depletion (CADD), and dbNSFP-Polyphen2-HDIV. Allele frequencies (AF) of those variants were determined using the QGP dataset, the genome aggregation database (gnomAD), Greater Middle East (GME), and the 1000 Genomes (1 KG) Project database.

### Molecular structure analysis using PyMol

The crystallized partial G6PD structure was retrieved from PDB (PDB ID: 2BHL [15]). Only non-synonymous variants that were not previously investigated in Doss et al. [10] and whose native residues were crystallized in 2BHL were analyzed. PyMol version 2.4.1 was used to model the 18 selected variants in order to visualize the potential structural changes they introduce to the native protein structure [16].

### Sanger sequencing

The novel variants identified in this study were confirmed by PCR and Sanger sequencing. The primers used are given in Additional 2: Table SA1 using the ABI 373 automated sequencer, and the data were analyzed using the Mutation Surveyor software (http://www.softgenetics.com/mutationSurveyor.html).

### G6PD deficiency assay

To investigate the functional impact of the novel *G6PD* variants identified, we used a quantitative assay to measure G6PD enzymatic activity in frozen red blood cells (RBCs) obtained from the variant carriers. The samples were tested for their enzyme activity at the diagnostic laboratories of Hamad Medical Cooperation (HMC), using G6PDH assay (RANDOX, cat. no. PD 410), following the manufacturer’s protocol. G6PD activity measured in units of enzyme activity was normalized for RBC count as per the following formula. The reference range used to determine deficiency is as per HMCs protocol (224.1–516.9 mU/10^9^ RBC), and this is based on the normal activity of the enzyme observed in hemizygous males from the Qatari population.

$${\text{G6PDH}}\,{\text{mU}}/10^{9} {\text{RBCs}}\, = \,{\text{G6PDH}}\,{\text{activity}}\,{\text{in}}\,{\text{mU}}/{\text{mL}}/{\text{RBC's}}\,{\text{count}}\,10^{6} /\upmu {\text{l}}.$$

## Results

### G6PD variants in the Qatari population

**Fig. 1:**
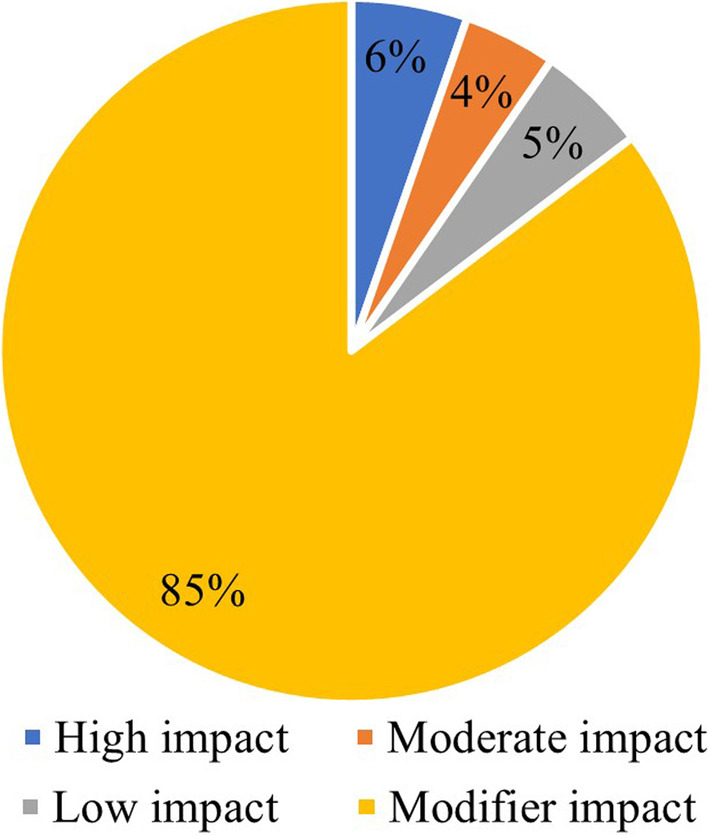
Percentage of the high-, moderate-, low-, and modifier-impact variants identified in/near *G6PD* gene

**Fig. 2:**
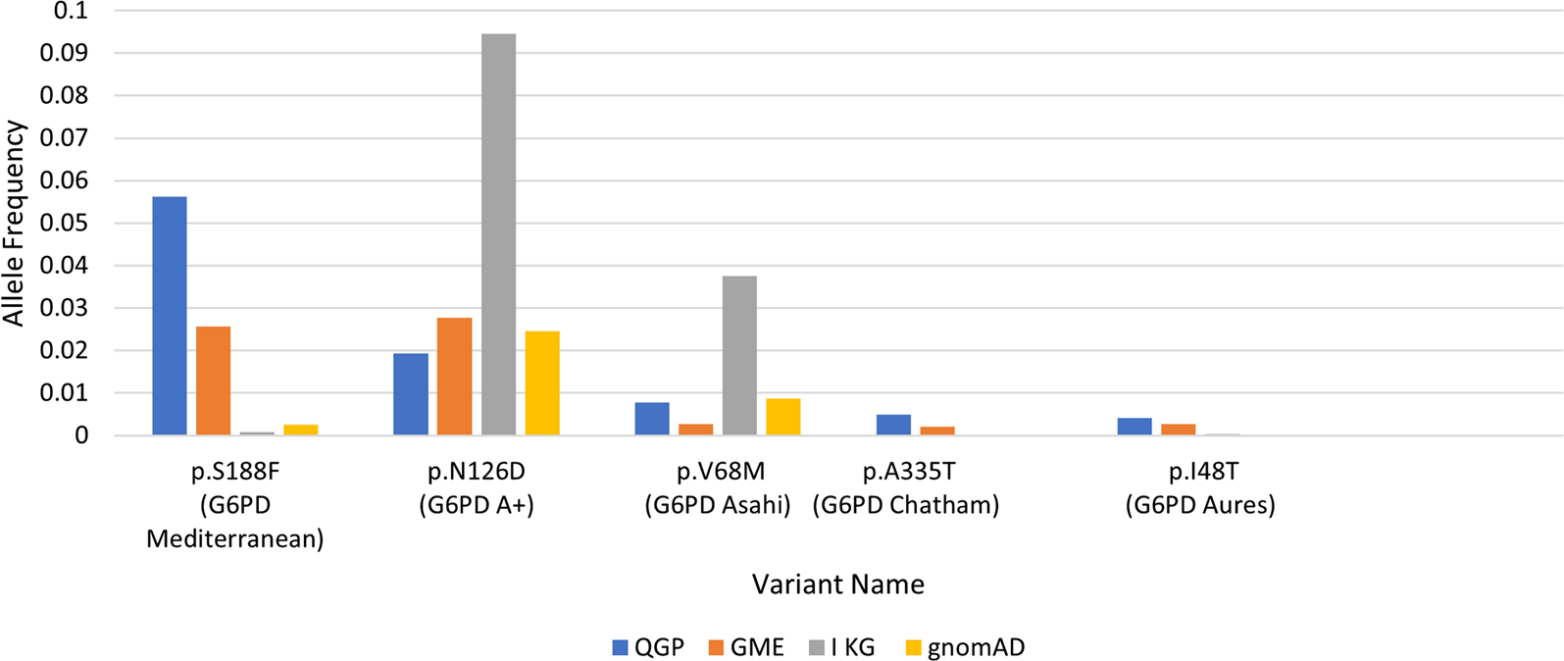
Frequencies of the five most common *G6PD*-causing variants in the Qatari population and their frequencies in QGP, GME, 1 KG, and gnomAD. G6PD Asahi and G6PD Chatham are high-impact variants, while G6PD Mediterranean, G6PD Aures, and G6PD A + are moderate-impact variants

**Fig. 3:**
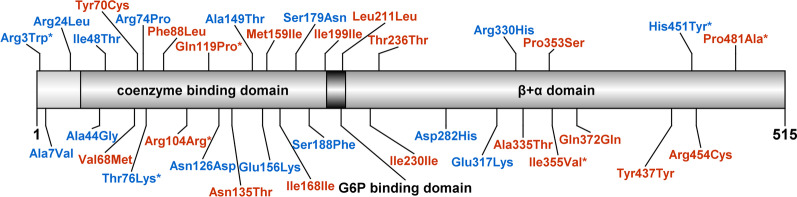
Linear G6PD structure showing the positions of the 36 reported G6PD mutations. High-impact variants are shown in red, while moderate-impact variants are shown in blue. Novel variants are denoted with an asterisk (*). For p.Met159Ile, two different genetic variants resulted in the the same amino acid change

We identified from the WGS data of 6,045 QGP participants 375 variants in/nearby the *G6PD* gene. These include 20 high-impact, 16 moderate-impact, 19 low-impact, and 320 modifier-impact variants (Fig. 1). We focused on the high-impact and moderate-impact variants (36 variants) as previously indicated in Sect. 2.3. The five most common variants seen in the Qatari population and their corresponding frequencies in other databases are indicated in Fig. 2. The high-impact and moderate- impact variants are indicated in a liner protein diagram in Fig. 3, constructed using DOG V 2.0.1 [17] based on the annotation mentioned in the literature [6, 15].

### Analysis of the high- and moderate-impact variants

We investigated 36 variants, including 20 high-impact and 16 moderate-impact variants. Of those, seven variants were novel (four high-impact and three moderate-impact variants). The frequencies of those variants and their CADD scores are given in Tables 1 and 2. Their predicted pathogenicity scores using SIFT, Polyphen, and dbNSFP-Polyphen2-HDIV are also presented as additional material in Additional file 2: Tables SA2 and SA3. The WHO classification for the well-established G6PDD-causing variants were also included when available (Additional file 2: Tables SA2 and SA3). The subpopulation frequencies of the 36 variants were also collected from QGP data for the six subpopulations in Qatar which are Peninsular Arabs (PAR) (17.4%), General Arabs (GAR) (38.2%), West Eurasian/Persians (WEP) (22.7%), Africans (AFR) (1.5%), South Asians (SAS) (0.6%), and Admixed (ADM) (19.5%) (Additional file 2: Table SA4).

**Table 1:** High-impact variants identified in the *G6PD* gene

Position	rs ID	Variant name	CDS substitution	Amino acid substitution	Exon	CADD score	HGMD	Frequency in QGP	Frequency in GME	Frequency in gnomAD
† 153760419			c.1441C > G	p.Pro481Ala	12	7.925		0.00008268	0.00139	
153760605	rs398123546	*G6PD* Union	c.1360C > T	p.Arg454Cys	11	34	DM	0.00008269		0.0001071
‡153760654	rs2230037	–	c.1311C > T	p.Tyr437Tyr	11	6.988	DM?	0.284687		0.169
153760953	rs2230036	–	c.1116G > A	p.Gln372Gln	10	4.741	–	0.00529188	0.00691	0.009705
† 153761006			c.1063A > G	p.Ile355Val	10	6.273		0.00041342		
153761012	rs137852333	*G6PD* Ierapetra	c.1057C > T	p.Pro353Ser	10	24.8	DM	0.00033074		
153761205	rs5030869	*G6PD* Chatham	c.1003G > A	p.Ala335Thr	9	27.3	DM	0.00504382	0.00207	0.0002306
153762312	rs782118135	–	c.708C > T	p.Thr236Thr	7	8.209	–	0.00024805		0.00001647
153762330	rs781917123	–	c.690C > T	p.Ile230Ile	7	13.01	–	0.00016537		0.0007578
153762564	rs781866029	–	c.633C > T	p.Leu211Leu	6	10.35	–	0.00008268		–
153762600	rs369516039	–	c.597C > T	p.Ile199 Ile	6	10.89	–	0.00008268		
153762693	rs782678149	–	c.504C > T	p.Ile168 Ile	6	12.96	–	0.00016537		0.00001647
153763391	rs370918918		c.477G > C	p.Met159Ile	5	18.24	DM?	0.00223251		0.001219
153763391	rs370918918		c.477G > A	p.Met159Ile	5	20.2		0.00016537		
153763464	rs782322505		c.404A > C	p.Asn135Thr	5	24.5	DM	0.00033074		0.000008237
† 153763512			c.356A > C	p.Gln119Pro	5	2.238		0.00008268		
† 153763556		–	c.312C > T	p.Arg104Arg	5	2.941	–	0.00016537		
153764155	rs781794862		c.264C > G	p.Phe88Leu	4	2.678		0.00008268		0.0001565
153764210	rs782090947		c.209A > G	p.Tyr70Cys	4	22.9	DM	0.00016537		0.000008237
153764217	rs1050828	*G6PD* Asahi	c.202G > A	p.Val68Met	4	27.2	DM	0.00785513	0.00275	0.009974

Novel variants are denoted with a dagger (†). Variants are described in relation to NM_001042351.3; those denoted with (‡) are described in relation to NM_001042351.1. CDS: coding sequence

**Table 2:** Moderate-impact variants identified in the *G6PD* gene

Position	rs ID	Variant name	CDS substitution	Amino acid substitution	Exon	CADD score	HGMD*	Frequency in QGP	Frequency in GME	Frequency in gnomAD
† 153760614	–	–	c.1351C > T	p.His451Tyr	11	25.6	–	0.00016537		
153761219	rs868950643	–	c.989G > A	p.Arg330His	9	7.666	–	0.0116587		
153761259	rs137852339	*G6PD* Kalyan	c.949G > A	p.Glu317Lys	9	22.6	DM	0.00090954		0.001664
153761811	rs137852318	*G6PD* Modena	c.844G > C	p.Asp282His	8	24.8	DM	0.00008268		0.000865
153762634	rs5030868	*G6PD* Mediterranean	c.563C > T	p.Ser188Phe	6	24.2	DM	0.0563089	0.02569	0.003097
153762661	rs281860640	*-*	c.536G > A	p.Ser179Asn	6	25.9	DM	0.00016537		
153763402	rs137852313	*G6PD* Ilesha	c.466G > A	p.Glu156Lys	5	8.019	DM	0.0010749		0.000313
153763423	rs782669677	–	c.445G > A	p.Ala149Thr	5	10.11	DM	0.00024805		0.00001647
153763492	rs1050829	*G6PD* A +	c.376A > G	p.Asn126Asp	5	0.037	DM	0.0194311	0.027759	0.029
† 153764192	–	–	c.227C > A	p.Thr76Lys	4	18.76	-	0.00008268		
153764198	–	–	c.221G > C	p.Arg74Pro	4	10.28	–	0.00107491		
153764371	rs76645461	*G6PD* Aures	c.143 T > C	p.Ile48Thr	3	18.09	DM	0.0038035	0.00276	0.000008237
153764383	rs78478128	*G6PD* Orissa	c.131C > G	p.Ala44Gly	3	28.9	–	0.00016537		0.000272
‡153775015	rs782006658	*–*	c.71G > T	p.Arg24Leu	1	12.05	–	0.00041377		0.001074
153775066	rs782150651	–	c.20C > T	p.Ala7Val	1	22.2	–	0.00049718		0.00003676
‡†153775079	–	–	c.7C > T	p.Arg3Trp	1	19.35	–	0.00024888		–

Novel variants are denoted with a dagger (†). Variants are described in relation to NM_001042351.3; those denoted with (‡) are described in relation to NM_000402.4. CDS: coding sequence

### Molecular visualization

**Fig. 4:**
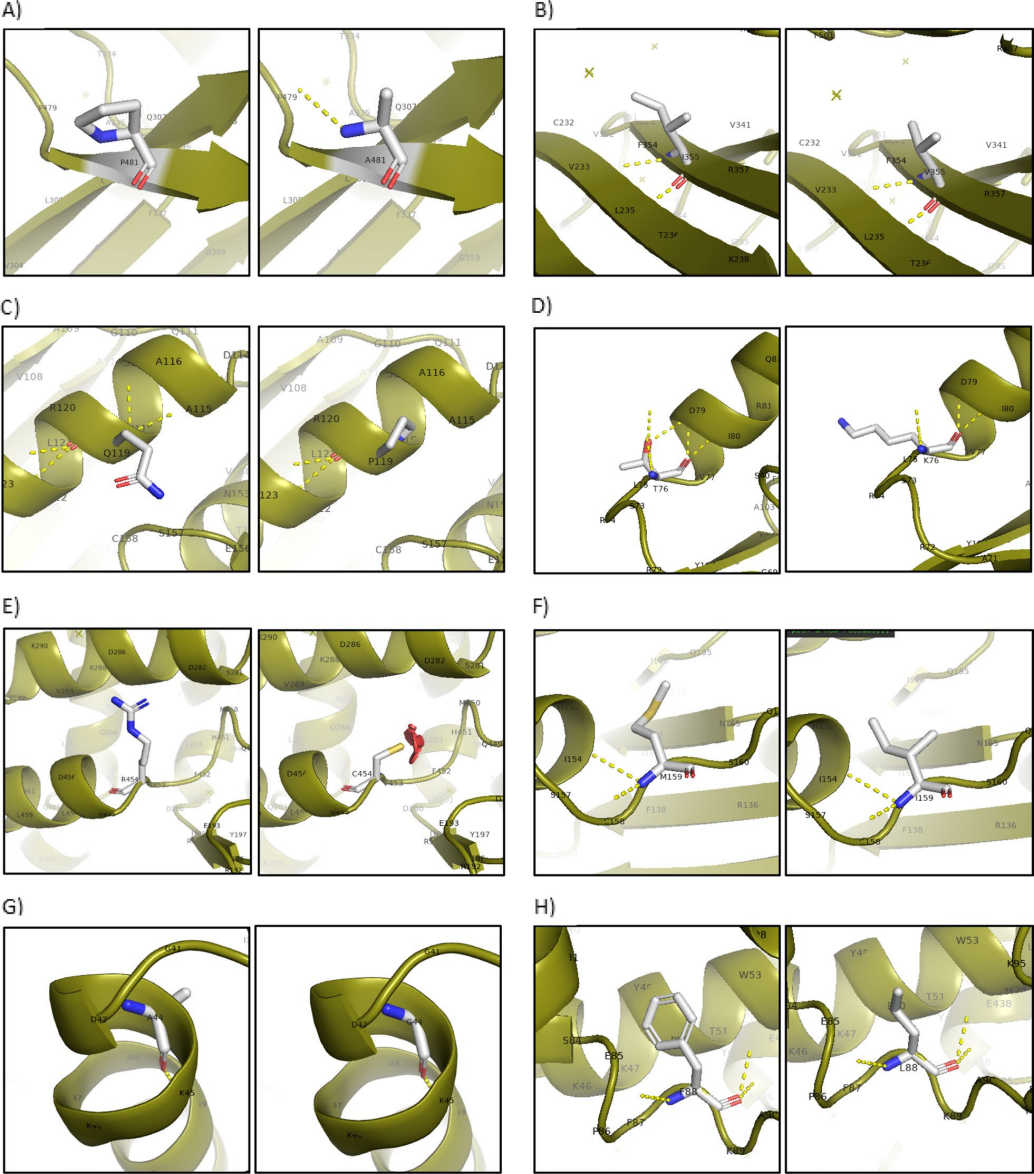
**a** p.Pro481Ala showing gain of polar contact with p479, **b** p.Ile355Val, **c** p.Gln119Pro showing loss of contacts with A115 and A116, **d** p.Thr76Lys showing loss of some polar contacts with D79, **e** p.Arg454Cys; a rotamer of this variant clashes with surrounding residues (red spheres), **f** p.Met159Ile, **g** p.Ala44Gly, H) p.Phe88Leu, **i** p.Tyr70Cys showing loss of contact with Q111, **j** p.Glu156Lys, where the native residue is substituted for the larger residue lysine, **k** p.Ala149Thr showing gain of polar contacts with V150, **l** p.Arg74Pro, where the native residue is substituted for the smaller residue, proline, **m** p.Arg330His, **n** p.Glu317Lys, **o** p.Asp282His showing gain of polar contacts with Q449 and R454, loss of contacts with R285; a rotamer of this variant also clashes with surrounding residues, **p** p.His451Tyr showing gain of polar contacts with Q449 and Q209, Q) Ala335Thr showing minor clash points with surrounding residues (green sphere), and **r** Pro353Ser showing gain of polar contacts with Gly351

In silico analysis using PyMol was preformed to reveal potential structural changes introduced by the identified variants. Modeled variants include six previously reported high-impact variants (p.Arg454Cys, p.Met159Ile, p.Phe88Leu, p.Pro353Ser, p.Ala335Thr, and p.Tyr70Cys), seven moderate-impact variants (p.Ala149Thr, p.Glu156Lys, p.Arg330His, p.Glu317Lys, p.Ala44Gly, p.Arg74Pro, and p.Asp282His), and five novel variants (p.Pro481Ala, p.Ile355Val, p.Gln119Pro, p.His451Tyr, and p.Thr76Lys). The variants p.Gln119Pro, p.Thr76Lys, p. Tyr70Cys, and p.Asp282His are predicted to cause loss of polar contacts, which could lead to the destabilization of the protein structure (Fig. 4c, d, i, o). The variants p.Pro481Ala, p.Ala149Thr, p.Asp282His, p.His451Tyr, and p.Pro353Ser resulted in gain of polar contacts, which could conversely over-stabilize the protein structure (Fig. 4a, k, o, p, r). Rotamers of p.Arg454Cys, p.Asp282His, and p.Ala335Thr were predicted to clash with surrounding residues, which could lead to steric hindrance in G6PD as shown in Fig. 4e, o, q. Finally, substitutions that greatly change the size of the native amino acid were also observed such as p.Glu156Lys and p.Arg74Pro shown in Fig. 4j and l, respectively.

### Enzyme activity assay

The seven novel variants were seen in a total of 14 participants (12 females and 2 males). Of those, all were G6PD deficient, except for two female participants who had G6PD levels that fall in the lower normal range (Table 3).

**Table 3:** Enzyme activity assay results for the carriers of the seven novel variants identified in the Qatari population

Variant position	Amnio acid change	Sample ID	Subject gender	Subject zygosity status	RBC count (× 10^6^/ul)	G6PDH (mU/mL)	G6PDH (mU/10^9^ RBC)	Interpretation*
153775079	p.Arg3Trp	QU000105000014	M	Hemizygous	6	1010	168	Deficient
		QU000105000006	F	Heterozygous	4.4	997	227	Normal
		QU000105000007	F	Heterozygous	5	1013	203	Deficient
153760614	p.His451Tyr	QU000105000011	F	Heterozygous	4.5	602	134	Deficient
		QU000105000010	F	Heterozygous	5.8	878	151	Deficient
153764192	p.Thr76Lys	QU000105000003	F	Heterozygous	4.4	700	159	Deficient
153760419	p.Pro481Ala	QU000105000001	F	Heterozygous	5.6	1075	192	Deficient
153761006	p.Ile355Val	QU000105000013	M	Hemizygous	4.2	733	175	Deficient
		QU000105000012	F	Heterozygous	4	1004	251	Normal
		QU000105000009	F	Heterozygous	4.2	725	173	Deficient
		QU000105000004	F	Heterozygous	4	526	132	Deficient
153763512	p.Gln119Pro	QU000105000002	F	Heterozygous	4.2	341	81	Deficient
153763556	P.Arg104Arg	QU000105000005	F	Heterozygous	5.3	668	126	Deficient
		QU000105000008	F	Heterozygous	3.9	678	174	Deficient

M: male, F: female

*G6PDH of 244 mU/10 ^9^ RBC or below is interpreted as deficient

## Discussion

This study is the first to investigate the genetic basis of G6PDD in Qatar. We used WGS data from 6045 phenotypically healthy QBB participants and identified p.Ser188Phe (*G6PD* Mediterranean), p.Asn126Asp (G6PD A+), p.Val68Met (G6PD Asahi), p.Ala335Thr (G6PD Chatham), and p.Ile48Thr (G6PD Aures) as the five most common disease-causing G6PD variants in our QBB cohort with AFs of 0.0563, 0.0194, 0.00785, 0.0050, and 0.00380, respectively. This is consistent with findings from the Arab world, in which p.Ser188Phe (*G6PD* Mediterranean), p.Asn126Asp (G6PD A+), p.Val68Met (G6PD Asahi), and p.Ile48Thr (*G6PD* Aures) were reported to be the most common *G6PD* variants in Arab G6PPD patients, with prevalence of 56.80%, 10.03%, 10%, and 5.51%, respectively [10]. The p.Tyr437Tyr (rs2230037) variant was the most common variant seen in this study with AF of 0.288. It has been described as a polymorphism in various studies [18, 19], a “benign” variant in ClinVar, and as potentially causative in some studies [20].

Studies conducted in the UAE, Jordan, Iraq, and Oman also reported relatively low prevalence (0.5–8.7%) of G6PD Chatham cases among G6PDD patients [21–24]. These similarities in G6PD variations observed across different Arab populations reflect the common ancestry and similar genetic background of Arabs. Moreover, Doss and Alasmar [10] report four variants to be exclusively found in the Arab world, namely p.Asp135Thr, p.Ser179Asn, p.Arg246Leu, and p.Glu307Pro. The variant p.Asp135Thr was observed in Egypt, Palestine, and Jordan [10, 22, 25], and we report it also here in our cohort with AF of 0.00033074. The variant p.Ser179Asn was reported in Palestine and was also seen in our study (AF = 0.00016537). On the other hand, the other two variants, p.Arg246Leu and p.Glu307Pro, which were initially seen in Tunisia, were not detected in our study.

We also investigated the frequencies of the 36 variants within the six distinct subpopulations that constitute the Qatari population. The most common variant identified in this study, G6PD Mediterranean, was predominantly seen in West Eurasian/Persians (WEP) subpopulation with AF of 0.134475, while in gnomAD it was mostly seen in South Asians subpopulation with AF of 0.0173. Three variants G6PD A +, G6PD Asahi, and G6PD Aures were mainly identified in Africans (AFR) with AFs of 0.173913, 0.0706522, and 0.0108696, respectively, while G6PD Chatham was mainly seen in WEP (AF = 0.0193149). In gnomAD, the frequencies and distributions of those variants among subpopulations are different; G6PD A + is mainly identified in Latinos (AF = 0.001130), G6PD Chatham in Europeans (AF = 000001232), G6PD Aures in East Asians (AF = 0.0001443), and G6PD Asahi in Africans (AF = 0.1164). This can be due to the fact that Arabs and Middle Eastern populations are underrepresented in gnomAD and other publicly available databases.

Conservation studies report three motifs in the G6PD protein to show particularly high conservation: these being the 198-RIDHYLGKE-206 motif which is required for the binding and catalysis of glucose-6-phosphate (G6P) [26], the 38-GASGDLA-44 motif (termed the nucleotide-binding fingerprint) which constitutes a region where the coenzyme binds, and finally 170-EKPxG-174 which is needed for the correct positioning of both the coenzyme and the substrate [15, 27, 28]. Only two variants were seen within these motifs in our study: the synonymous variant p.Ile199Ile with AF of 0.000082685 and the *G6PD* Orissa variant (p.Ala44Gly) with AF of 0.000165371. Their low frequencies further suggest that variations within these regions might not be tolerated. Finally, most of the analyzed variants (22%) were found to be within exon 5, suggesting that this exon is a hot spot for missense variants. We also identified variants in exons 6, 10, and 13, which are reported to be within the substrate-binding domain of G6PD (Additional file 2: Tables SA2 and SA3) [29].

This study identified seven novel G6PD variants (not seen in other variants databases) and investigated their impact on G6PD activity. These were classified as class III variants since they resulted in enzymatic activity between the range of 10 and 60% [5]. Table 3 shows a summary of the G6PD activity assay findings for the novel variants. Among the high-impact novel variants, the p.Pro481Ala variant, which substitutes the polar amino acid proline with the non-polar amino acid alanine, is predicted to introduce additional polar contacts with the nearby Pro479 (Fig. 4a). The variant has a relatively low CADD score of 7.925 and is predicted to be “benign” and “tolerated” in Polyphen and SIFT, respectively. This variant was identified in one heterozygous female which was G6PD deficient based on the enzymatic activity test, which contradicts the predication and might also cause deficiency due to X-inactivation skewness in favor of the deficient allele in the female carrier. In the second novel variant, p.Ile355Val, both the native and mutant amino acids are non-polar and are of similar size (Fig. 4b). No loss or gain of polar contacts is observed after molecular visualization which correlates with their annotation as “benign” and “tolerated” in Polyphen and SIFT, respectively. The biochemical activity test showed that three participants (one hemizygous male and two heterozygous females) carrying this variant were found to be G6PD deficient. However, another heterozygous female appeared to have G6PD activity in the lower normal range (251 mU/10^9^ RBC). The variation in the G6PD status observed among the heterozygous females from deficient to normal is likely to reflect variations in the ratios of G6PD deficient to G6PD normal RBCs as a result of variations in X-inactivation patterns during early embryogenesis [30, 31]. The p.Gln119Pro is the third high-impact novel variant. Here, the polar-uncharged amino acid glycine is substituted for the non-polar amino acid, proline. This was predicted to cause loss of polar contacts with two of the nearby amino acids, Ala115 and Ala116, indicating loss of stability (Fig. 4e, f). The variant was, however, predicted to be “benign” and “tolerated” by Polyphen and SIFT, respectively. One heterozygous female carried this variant, and she was tested to have deficient G6PD activity. Finally, p.Arg104Arg is a synonymous variant and it has a low CADD score of 2.941, but was classified as high-impact variant by SnpEff which takes into account the location of the variant as well as its role in interaction [14]. In agreement with SNPeff prediction, the female carrier of this variant was G6PD deficient.

Among the moderate-impact novel variants, the polar uncharged threonine is replaced with the polar, positively charged, and much larger lysine in the p.Thr76Lys variant. This is predicted to cause loss of some polar contacts with the neighboring Asp79, potentially destabilizing the enzyme (Fig. 4g, h). The variant is classified as “damaging” and “possibly damaging” in Polyphen and SIFT, respectively. This variant was seen in one female participant (heterozygous) within our study who was reported as G6PD deficient. In the second moderate-impact novel variant, p.Arg3Trp, the positively charged, polar amino acid, arginine is substituted for the aromatic amino acid tryptophan. This substitution was predicted to be damaging by SIFT, and it had a relatively high CADD score of 19.35. Of the three participants tested for this variant, two were found to be deficient, while one heterozygous female showed low normal G6PD activity (227 mU/10^9^ RBC). In the final moderate-impact novel variant, p.His451Tyr, the polar amino acid histidine is substituted for the aromatic amino acid tyrosine. It was predicted to be “probably damaging” and “tolerated” by Polyphen and SIFT, respectively, and has the highest CADD score of 25 among the novel variants identified. The two participants carrying this variant (heterozygous females) were found to be G6PD deficient, supporting their causality.

## Conclusion

In summary, this study revealed novel G6PDD variants and elucidated their genotype–phenotype correlation. In addition, we determined the frequencies of some common G6PD variants in the Qatari population. Our work highlights the importance of investigating understudied populations in providing novel insights about disease pathogenesis.

## Supplementary Information

**Additional file 1**: **Figure S1.** Principal component analysis plot for the genomes used in this study.

**Additional file 2**: Table SA1. Primers used to confirm the novel G6PD variants using Sanger sequencing. Table A2. High-impact variants identified in the G6PD gene. Table A3. Moderate-impact variants identified in the G6PD gene. Table A4. Subpopulation frequencies of the identified high- and moderate-impact variants.

## Data Availability

The informed consent given by the study participants does not cover posting of participant level phenotype and genotype data of Qatar Biobank/Qatar Genome Project in public databases. However, access to QBB/QGP data can be obtained through an established ISO-certified process by submitting a project request at https://www.qatarbiobank.org.qa/research/how-to-apply which is subject to approval by the QBB IRB Committee. The datasets supporting the conclusions of this article are included within the article and its additional file.

## Abbreviations

ADM: Admixed
AF: Allele frequency
AFR: Africans
CADD: Combined annotation-dependent depletion
ExAC: Exome Aggregation Consortium
GME: The Greater Middle East database
GAR: General Arab
G6PD: Glucose-6-phospate dehydrogenase
G6PDD: Glucose-6-phosphate dehydrogenase deficiency
gnomAD: Genome aggregation database
HMC: Hamad Medical Cooperation
HGMD: Human Gene Mutation Database
NADPH: Nicotinamide adenine dinucleotide phosphate
QBB: Qatar Biobank
QGP: Qatar Genome Programme
RBC: Red blood cell
RS ID: Reference SNP cluster ID
ASA: South Asians
SIFT: Sorting intolerant from tolerant
WEP: West Eurasian/Persians
WGS: Whole-genome sequencing
WHO: World Health Organization
VCF: Variant call format
PAR: Peninsular Arab
Polyphen: Prediction of functional effects of human nsSNPs
1 Kg: 1000 Genomes Project database
