# Supplementary figures and images for: Seven novel glucose-6-phosphate dehydrogenase (G6PD) deficiency variants identified in the Qatari population
Source: Hum Genomics. 2021 Oct 7;15:61. doi: 10.1186/s40246-021-00358-9 (PMC8499492; doi:10.1186/s40246-021-00358-9)

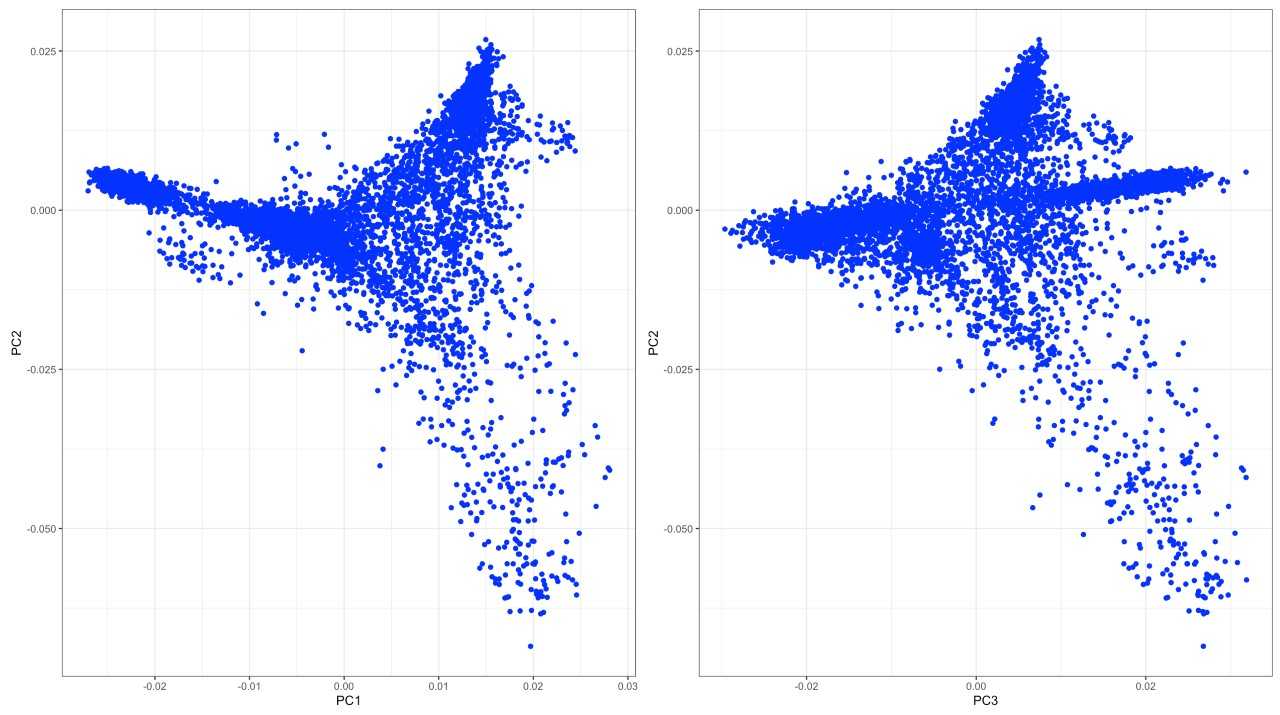

Supplement: Supplementary file 1 — Additional file 1: Figure S1. Principal component analysis plot for the genomes used in this study. [file 40246_2021_358_MOESM1_ESM.jpeg]
