# Supplementary material for: Seven novel glucose-6-phosphate dehydrogenase (G6PD) deficiency variants identified in the Qatari population
Source: Hum Genomics. 2021 Oct 7;15:61. doi: 10.1186/s40246-021-00358-9 (PMC8499492; doi:10.1186/s40246-021-00358-9)
Supplement: Supplementary file 2 — Additional file 2: Table SA1. Primers used to confirm the novel G6PD variants using Sanger sequencing. Table A2. High-impact variants identified in the G6PD gene. Table A3. Moderate-impact variants identified in the G6PD gene. Table A4. Subpopulation frequencies of the identified high- and moderate-impact variants. [file 40246_2021_358_MOESM2_ESM.docx]

**Table A1.** Primers used to confirm the novel G6PD variants using sanger sequencing.

| **Variant position** | **Amino acid substitution** | **Product size, bp** | **Primer** | **Sequence** | **Melting temperature,** **°C** |
| --- | --- | --- | --- | --- | --- |
| 153775079 | p.Arg3Trp | 496 | Forward | AATTGGGGATGCAGAGCA | 60.16 |
|  |  |  | Reverse | GGTCGGCAAGTCCCCTTC | 62.97 |
| 153760614‎ | p.His451Tyr | 375 | Forward | TGGCATCAGCAAGACACTCT | 59.58 |
| 153760419 | p.Pro481Ala |  | Reverse | TGAGGTAGCTCCACCCTCAC | 60.26 |
| 153764192‎ | p.Thr76Lys | 397 | Forward | GTGACCTGGCCAAGAAGAAG | 59.84 |
|  |  |  | Reverse | AATGGGGGTCTCAAGGAAGT | 59.79 |
| 153761006 | p.Ile355Val | 389 | Forward | GAGAATGAGAGGTGGGATGG | 59.46 |
|  |  |  | Reverse | ATCTGTTGCCGTAGGTCAGG | 60.13 |
| 153763512 | p.Gln119Pro | 382 | Forward | AGGGAAGACCCTCCACTCC | 60.45 |
| 153763556 | P.Arg104Arg |  | Reverse | CATAGAGTGGTGGGAGCACTG | 60.72 |

**Table A 2.** High impact variants identified in the G6PD gene.

| **#** | **Position** | **rs ID** | **Exon** | **CDS substitution** | **Amino acid substitution** | **variant name** | **Variant type** | **Allele count** | **HGMD** | **Polyphen** | **SIFT** | **dbNSFP-Polyphen2  -HDIV Score** | **ClinVar** | **Frequency in 1kg** |
| --- | --- | --- | --- | --- | --- | --- | --- | --- | --- | --- | --- | --- | --- | --- |
| 1. | **†** 153760419 | . | 12 | c.1441C>G | p.Pro481Ala | *.* | Missense | 1 | - | Benign (0) | Tolerated (0.17) | 0,0.002 | . | . |
| 2. | 153760605 | rs398123546 | 11 | c.1360C>T | p.Arg454Cys | *G6PD Union* | Missense | 1 | DM | Probably damaging (0.997) | Damaging (0) | 1 | Pathogenic | 0.0005298 |
| 3. | **†** 153761006 | . | 10 | c.1063A>G | p.Ile355Val | *.* | Missense | 5 | - | Benign (0.017) | Tolerated (0.18) | 0.001,0.007 | . | . |
| 4. | 153761012 | rs137852333 | 10 | c.1057C>T | p.Pro353Ser | *G6PD Ierapetra* | Missense | 4 | DM | Probably damaging (0.961) | Damaging (0.01) | 0.999 | Conflicting | - |
| 5. | 153761205 | rs5030869 | 9 | c.1003G>A | p.Ala335Thr | *G6PD Chatham* | Missense | 61 | DM | Possibly damaging (0.569) | Damaging (0) | 0.961,0.985 | Pathogenic/other | . |
| 6. | 153763391 | rs370918918 | 5 | c.477G>C | p.Met159Ile | *-* | Missense | 27 | DM? | Benign (0.268) | Tolerated (0.15) | 0.532,0.424 | Benign. | 0.0015894 |
| 7. | 153763391 | rs370918918 | 5 | c.477G>A | p.Met159Ile | *-* | Missense | 2 | - | Benign (0.268) | Tolerated (0.15) | 0.532,0.424 | - | - |
| 8. | 153763464 | rs782322505 | 5 | c.404A>C | p.Asn135Thr | *-* | Missense | 4 | DM | Probably damaging (0.992) | Damaging (0.02) | 0.998,0.997 | - | - |
| 9. | **†** 153763512 | . | 5 | c.356A>C | p.Gln119Pro | *-* | Missense | 1 | - | Benign (0.153) | Tolerated (0.16) | 0.083,0.207 | - | - |
| 10. | 153764155 | rs781794862 | 4 | c.264C>G | p.Phe88Leu | *-* | Missense | 1 | - | Benign (0.003) | Tolerated (1) | 0,0.001 | - | 0.0005298 |
| 11. | 153764210 | rs782090947 | 4 | c.209A>G | p.Tyr70Cys | *-* | Missense | 2 | DM | Probably damaging (0.992) | Damaging (0) | 1 | - | - |
| 12 | 153764217 | rs1050828 | 4 | c.202G>A | p.Val68Met | *G6PD Asahi* | Missense | 95 | DM | Probably damaging (0.944) | Damaging (0.02) | 0.999 | Drug response | 0.0376 |
| 13 | ‡153760654 | rs2230037 | 11 | c.1311C>T | p.Tyr437Tyr | *-* | synonymous | 3443 | DM? | A | - | - | Benign/Likely benign | 0.2159 |
| 14 | 153760953 | rs2230036 | 10 | c.1116G>A | p.Gln372Gln | *-* | synonymous | 64 | - | - | - | - | Benign | 0.0334 |
| 15 | 153762312 | rs782118135 | 7 | c.708C>T | p.Thr236Thr | *-* | synonymous | 3 | - | - | - | - | - | - |
| 16 | 153762330 | rs781917123 | 7 | c.690C>T | p.Ile230Ile | *-* | synonymous | 2 | - | - | - | - | Benign | 0.0007947 |
| 17 | 153762564 | rs781866029 | 6 | c.633C>T | p.Leu211Leu | *-* | synonymous | 1 | - | - | - | - | - | - |
| 18 | 153762600 | rs369516039 | 6 | c.597C>T | p.Ile199 Ile | *-* | synonymous | 1 | - | - | - | - | - | - |
| 19 | 153762693 | rs782678149 | 6 | c.504C>T | p.Ile168 Ile | *-* | synonymous | 2 | - | - | - | - | - | - |
| 20 | **†** 153763556 | . | 5 | c.312C>T | p.Arg104Arg | *-* | synonymous | 2 | *-* | - | - | - | - | - |

Novel variants are denoted with a dagger (**†**). Variants are described in relation to NM_001042351.3, those denoted with (‡) are described in relation to NM_001042351.1. CDS: coding sequence; DM: disease-mutation; DM?; potential disease-mutation.

**Table A 3.** Moderate impact variants identified in the G6PD gene.

| **#** | **Position** | **rs ID** | **Exon** | **CDS substitution** | **Amino acid substitution** | **Variant name** | **Variant**  **Type** | **Allele count** | **HGMD** | **Polyphen** | **SIFT** | **dbNSFP-Polyphen2  -HDIV Score** | **ClinVar** | **Frequency in 1kg** |
| --- | --- | --- | --- | --- | --- | --- | --- | --- | --- | --- | --- | --- | --- | --- |
| 1 | ‎153762661‎ | rs281860640‎ | 6 | c.536G>A | p.Ser179Asn | *-* | Missense | ‎2‎ | DM | Probably damaging (0.959) | Damaging (0) | 0.9,0.928 | . | . |
| 2 | ‎‡153775015‎ | rs782006658‎ | 1 | c.71G>T | p.Arg24Leu | *-* | Missense | ‎5‎ | *-* | - | Tolerated (0.32) | 0 | - | 0.0010596 |
| 3 | ‎153761811‎ | rs137852318‎ | 8 | c.844G>C | p.Asp282His | *G6PD Modena* | Missense | ‎1‎ | DM | Probably damaging (0.967) | Damaging (0.03) | 0.995,0.993 | Pathogenic | 0.0002649 |
| 4 | ‎153762634‎ | rs5030868 ‎ | 6 | c.563C>T | p.Ser188Phe | *G6PD Mediterranean* | Missense | ‎ 681 ‎ | DM | Benign (0.029) | Damaging (0.03) | 0.022,0.018 | Pathogenic | 0.0007947 |
| 5 | ‎153763492‎ | rs1050829 ‎ | 5 | c.376A>G | p.Asn126Asp | *G6PD A+* | Missense | ‎ 235 ‎ | DM | Benign (0.001) | Tolerated (0.94) | 0 | Conflicting | 0.0946 |
| 6 | ‎153763402‎ | rs137852313‎ | 5 | c.466G>A | p.Glu156Lys | *G6PD Ilesha* | Missense | ‎13‎ | DM | Benign (0.024) | Tolerated (0.78) | 0.005,0.028 | Conflicting | 0.0002649 |
| 7 | ‎153764198‎ | ‎-‎ | 4 | c.221G>C | p.Arg74Pro | *-* | Missense | ‎13‎ | *-* | Benign (0.009) | Tolerated (0.21) | 0 | - | - |
| 8 | ‎153764383‎ | rs78478128‎ | 3 | c.131C>G | p.Ala44Gly | *‎G6PD Orissa* | Missense | ‎2‎ | *-* | Probably damaging (0.996) | Damaging (0) | 0.998 | Conflicting | 0.0002649 |
| 9 | ‎153761219‎ | rs868950643‎ | 9 | c.989G>A | p.Arg330His | *‎-‎* | Missense | ‎141 | *-* | Benign (0.003) | Tolerated (0.14) | 0 | - | . |
| 10 | ‎‡153775066‎ | rs782150651‎ | 1 | c.20C>T | p.Ala7Val | *‎-‎* | Missense | ‎6‎ | *-* | - | Tolerated (0.48) | 0.435 | - | 0.0002649 |
| 11 | ‎‡**†** 153775079‎ | ‎-‎ | 1 | c.7C>T | p.Arg3Trp | *‎-‎* | Missense | ‎3 | *-* | - | Damaging (0) | 0 | - | . |
| 12 | **‎†** 153760614‎ | ‎-‎ | 11 | c.1351C>T | p.His451Tyr | *‎-‎* | Missense | ‎2‎ | *-* | Probably damaging (0.974) | Tolerated (0.12) | 0.999,1 | - | . |
| 13 | ‎**†** 153764192‎ | ‎-‎ | 4 | c.227C>A | p.Thr76Lys | *‎-‎* | Missense | ‎1‎ | *-* | Possibly damaging (0.465) | Damaging (0.05) | 0.92,0.768 | - | . |
| 14 | 153761259 | ‎ s137852339 | 9 | c.949G>A | p.Glu317Lys | *‎ G6PD Kalyan* | Missense | 11 | DM | Benign (0.388) | Tolerated (0.28) | 0.837,0.804 | Conflicting | 0.0021192 |
| 15 | 153763423 | ‎ rs782669677 | 5 | c.445G>A | p.Ala149Thr | *‎-‎* | Missense | 3 | DM | Benign (0.007) | Tolerated (0.64) | 0.001 | - | - |
| 16 | 153764371 | rs76645461 | 3 | c.143T>C | p.Ile48Thr | *‎ G6PD Aures* | Missense | 46 | DM | Possibly damaging (0.009) | Tolerated (0.6) | 0.985,0.994 | Conflicting | 0.0002649 |

Variants are described in relation to NM_001042351.3, those denoted with (‡) are described in relation to NM_000402.4. Novel variants are denoted with a dagger (**†**). CDS: coding sequence; DM: disease-mutation; DM?; potential disease-mutation.

| **Table A 4.** Sub-population frequencies of the identified high and moderate impact variants. |  |  |  |  |  |  |  | |  |  | **Sub-population frequencies** | | | |  |
| --- | --- | --- | --- | --- | --- | --- | --- | --- | --- | --- | --- | --- | --- | --- | --- |
| # | **Position** | **rs ID** | **Amino acid**  **substitution** | **Variant name** | **Frequency in QGP** | **GAR** | | **WEP** | | | | **AFR** | **SAS** | **ADM** | **PAR** |
|  | **†** 153760419 | . | p.Pro481Ala | - | 0.00008268 | 0 | | 0 | | | | 0 | 0 | 0.00042372 | 0 |
|  | 153760605 | rs398123546 | p.Arg454Cys | *G6PD Union* | 0.00008269 | 0.000216357 | | 0 | | | | 0 | 0 | 0 | 0 |
|  | **†** 153760614 | . | p.His451Tyr | - | 0.00016537 | 0.000432713 | | 0 | | | | 0 | 0 | 0 | 0 |
|  | §153760654 | rs2230037 | p.Tyr437Tyr | - | 0.284687 | 0.235396 | | 0.380831 | | | | 0.336957 | 0.342105 | 0.313136 | 0.229087452 |
|  | 153760953 | rs2230036 | p.Gln372Gln | - | 0.00529188 | 0.00367806 | | 0.00291545 | | | | 0.0597826 | 0 | 0.0118644 | 0 |
|  | **†** 153761006 | . | p.Ile355Val | - | 0.00041342 | 0.00108178 | | 0 | | | | 0 | 0 | 0 | 0 |
|  | 153761012 | rs137852333 | p.Pro353Ser | *G6PD Ierapetra* | 0.00033074 | 0 | | 0.00145773 | | | | 0 | 0 | 0 | 0 |
|  | 153761205 | rs5030869 | p.Ala335Thr | *G6PD Chatham* | 0.00504382 | 0.00021635 | | 0.0193149 | | | | 0.0108696 | 0 | 0.00211864 | 0 |
|  | 153761219 | rs868950643 | p.Arg330His | *‎-‎* | 0.0116587 | 0.00973605 | | 0 | | | | 0.00543478 | 0 | 0.00423729 | 0.04039924 |
|  | 153761259 | rs137852339 | p.Glu317Lys | *‎ G6PD_Kalyan* | 0.00090954 | 0.0015145 | | 0 | | | | 0 | 0.0263158 | 0.000847458 | 0 |
|  | 153761811 | rs137852318 | p.Asp282His | *G6PD Modena* | 0.00008268 | 0.00021635 | | 0 | | | | 0 | 0 | 0 | 0 |
|  | 153762312 | rs782118135 | p.Thr236Thr | *-* | 0.000248057 | 0 | | 0 | | | | 0 | 0 | 0 | 0.001425856 |
|  | 153762330 | rs781917123 | p.Ile230Ile | *-* | 0.000165371 | 0 | | 0.000364431 | | | | 0 | 0 | 0.000423729 | 0 |
|  | 153762564 | rs781866029 | p.Leu211Leu | *-* | 0.000082685 | 0.00021635 | | 0 | | | | 0 | 0 | 0 | 0 |
|  | 153762600 | rs369516039 | p.Ile199 Ile | *-* | 0.000082685 | 0 | | 0 | | | | 0 | 0 | 0.00042372 | 0 |
|  | 153762634 | rs5030868 | p.Ser188Phe | *G6PD Mediterranean* | 0.0563089 | 0.0266119 | | 0.134475 | | | | 0.0108696 | 0.0131579 | 0.0783898 | 0.000475285 |
|  | 153762661 | rs281860640 | p.Ser179Asn | *-* | 0.00016537 | 0 | | 0 | | | | 0.0108696 | 0 | 0 | 0 |
|  | 153762693 | rs782678149 | p.Ile168 Ile | *-* | 0.00016537 | 0.000432713 | | 0 | | | | 0 | 0 | 0 | 0 |
|  | 153763391 | rs370918918 | p.Met159Ile (c.477G>C) | - | 0.00223251 | 0.00086542 | | 0.00182216 | | | | 0 | 0.0131579 | 0.00720339 | 0 |
|  | 153763391 | rs370918918 | p.Met159Ile (c.477G>A) |  | 0.00016537 | 0 | | 0 | | | | 0 | 0 | 0.00084745 | 0 |
|  | 153763402 | rs137852313 | p.Glu156Lys | *G6PD_Ilesha* | 0.00107491 | 0 | | 0.000728863 | | | | 0 | 0 | 0.00466102 | 0 |
|  | 153763423 | rs782669677 | p.Ala149Thr | *‎-‎* | 0.00024805 | 0.000216357 | | 0 | | | | 0.00543478 | 0 | 0.00042372 | 0 |
|  | 153763464 | rs782322505 | p.Asn135Thr | *-* | 0.000330743 | 0.00064907 | | 0.000364431 | | | | 0 | 0 | 0 | 0 |
|  | 153763492 | rs1050829 | p.Asn126Asp | *G6PD A+* | 0.0194311 | 0.0103851 | | 0.0156706 | | | | 0.173913 | 0 | 0.0415254 | 0.006653992 |
|  | **†** 153763512 | . | p.Gln119Pro | *-* | 0.0000826856 | 0 | | 0 | | | | 0 | 0 | 0.00042372 | 0 |
|  | **†** 153763556 | . | p.Arg104Arg | *-* | 0.000165371 | 0 | | 0 | | | | 0 | 0 | 0.00084745 | 0 |
|  | 153764155 | rs781794862 | p.Phe88Leu | *-* | 0.000082685 | 0 | | 0 | | | | 0 | 0 | 0.00042372 | 0 |
|  | **†** 153764192 | . | p.Thr76Lys | *‎-‎* | 0.000082685 | 0.00021635 | | 0 | | | | 0 | 0 | 0 | 0 |
|  | 153764198 | . | p.Arg74Pro | *-* | 0.00107491 | 0 | | 0 | | | | 0 | 0 | 0 | 0.006178707 |
|  | 153764210 | rs782090947 | p.Tyr70Cys | *-* | 0.00016537 | 0 | | 0.000728863 | | | | 0 | 0 | 0 | 0 |
|  | 153764217 | rs1050828 | p.Val68Met | *G6PD Asahi* | 0.00785513 | 0.00108178 | | 0.013484 | | | | 0.0706522 | 0 | 0.0135593 | 0.003802281 |
|  | 153764371 | rs76645461 | p.Ile48Thr | *‎ G6PD_Aures* | 0.00380354 | 0.00757248 | | 0 | | | | 0.0108696 | 0 | 0.0038135 | 0 |
|  | 153764383 | rs78478128 | p.Ala44Gly | *‎G6PD Orissa* | 0.000165371 | 0 | | 0 | | | | 0 | 0.0263158 | 0 | 0 |
|  | ‡153775015 | rs782006658 | p.Arg24Leu | *-* | 0.00041377 | 0.000432713 | | 0 | | | | 0 | 0.0131579 | 0.000847458 | 0 |
|  | ‡153775066 | rs782150651 | p.Ala7Val | *‎-‎* | 0.000497183 | 0.000866551 | | 0 | | | | 0.010989 | 0 | 0 | 0 |
|  | ‡**†** 153775079 | . | p.Arg3Trp | *‎-‎* | 0.00024888 | 0 | | 0 | | | | 0.0164835 | 0 | 0 | 0 |

Novel variants are denoted with a dagger (**†)**. Variants are described in relation to NM_001042351.3, those denoted with (‡) are described in relation to NM_000402.4 while those denoted with (§) are described in relation to NM_001042351.1. GAR: Common Arabs cluster; WEP: Western Eurasians/Persians; AFR: East Africans; SAS: South Asians; ADM: Admixed; PAR: Peninsular Arabs.
